# Comparison of the therapeutic performance of macrolide antibiotics on macrolide-resistant or macrolide-susceptible Mycoplasma pneumoniae pneumonia children

**DOI:** 10.3389/fped.2026.1744694

**Published:** 2026-05-18

**Authors:** Yi-Qing Qiu, Xiao-Qian Chen

**Affiliations:** Department of Pediatrics, The First People's Hospital of Foshan, Foshan, Guangdong Province, China

**Keywords:** 23S rRNA, children, macrolide, macrolide-resistant Mycoplasma pneumoniae, Mycoplasma pneumoniae, refractory Mycoplasma pneumoniae pneumonia

## Abstract

**Background:**

This research aims to investigate the performance of macrolide antibiotics for the treatment of Mycoplasma pneumoniae pneumonia (MPP) in children with or without Mycoplasma pneumoniae (MP) resistance genes.

**Methods:**

This retrospective study enrolled pediatric MPP patients admitted to the Department of Pediatrics at the hospital between January 2023 and December 2023. Macrolide resistance genes in MP were tested using throat swab and bronchoalveolar lavage fluid samples, and patients were classified into the macrolide-resistant MP (MRMP) group and the macrolide-susceptible MP (MSMP) group based on the results of v-domain point mutations. After sampling, all patients received standard macrolide treatment. The baseline characteristics and laboratory examination results were documented and compared.

**Results:**

A total of 217 MPP patients were included, among these patients, 188 with v-domain point mutations (all A2063 G point mutation) were classified as MRMP (99 males, 5.84 ± 2.81 years) with an MRMP rate of 86.6%, the remaining 29 were classified as MSMP (14 males, 6.79 ± 4.08 years). No significant difference in baseline characteristics was observed between the patients of the two groups. While the MP DNA load was higher in both throat swabs (3.97 ± 0.64 vs. 3.65 ± 0.77; *P* = 0.039) and bronchoalveolar lavage fluid (4.11 ± 0.58 vs. 3.18 ± 0.77; *P* = 0.001) samples obtained from MRMP patients, the duration of macrolide therapy (7.66 ± 1.95 vs. 6.38 ± 2.37 days, *P* = 0.002) were also longer in MRMP patients. The incidence of severe Mycoplasma pneumoniae pneumonia (SMPP) was 5.8% in the MRMP group and 17.2% in the MSMP group (*P* = 0.071).

**Conclusion:**

MP DNA load and the duration of macrolide use are higher in MRMP patients, while no significant difference was observed between the prevalence of adverse events and clinical characteristics after treatment, indicating that macrolide treatment demonstrates comparable clinical effectiveness in both MRMP and MSMP patients. However, the association between MP DNA load and duration of macrolide use should be further evaluated.

## Introduction

*Mycoplasma pneumoniae* (MP) is a common pathogen causing pediatric Mycoplasma pneumoniae pneumonia (MPP), which is one type of the commonly observed community-acquired pneumonia (CAP), and accounts for approximately 10% to 40% of childhood CAP cases (1). Although MP infection is often considered mild and self-limiting (2), it is primarily transmitted through the respiratory tract, with cyclical pandemics occurring every 3 to 5 years (3). Following the relaxation of COVID-19 prevention policies, the resurgence of MP has raised significant public health concerns.

In the absence of a cell wall, MP is naturally resistant to antibiotics acting on the cell wall. Hence, antibiotics for the treatment of MP infections mainly include drugs that inhibit protein synthesis (including macrolides and tetracyclines) and restrain DNA replication (like fluoroquinolones) (3). However, due to limited safety data for tetracyclines and fluoroquinolones in children, their use is restricted (4). Macrolides remain the primary treatment for pediatric MP infections (5). However, the increasing clinical use of molecular diagnostics has contributed to a rise in macrolide-resistant MP (MRMP) strains (6). MRMP poses significant challenges, including treatment difficulties, increased complications, higher medical costs, and reduced quality of life (7). While asymptomatic MRMP infections are rare, macrolide resistance is a critical risk factor for MRMP (8). In recent years, the incidences of MRMP in different cities in China have mostly ranged between 70% and 92% (6, 9, 10). However, the 2024 expert consensus on the diagnosis and treatment of macrolide-resistant Mycoplasma pneumoniae pneumonia in children pointed out that Macrolide antibiotics are the first choice for treating MPP, while newer tetracycline antibiotics are also recommended for children >8 years (11).

In recent years, severe (SMPP) and refractory MPP (RMPP) cases have become more frequent, complicating clinical management. RMPP is defined as pneumonia that persists or worsens after 7 or more days of macrolide treatment, often accompanied by extrapulmonary symptoms such as pericarditis, central nervous system infections, arthritis, hemolytic anemia, and multi-organ failure (2, 3). Importantly, RMPP represents a treatment-response–defined subgroup within the broader spectrum of severe or progressive MPP. While MRMP infection has been proposed as a contributing factor to RMPP, excessive host immune response is also considered a critical determinant of disease severity (12). These factors can lead to poor clinical outcomes and severe complications (13). Macrolide resistance arises from specific mutations in the 23S rRNA gene's V region, particularly at the A2063 or A2064 loci, where adenine is replaced by cytosine, guanine, or thymine (14, 15). However, studies on the comparison of macrolide performance on MRMP and MSMP child populations are still lacking. Choi et al. reported that the children in the MRMP group had a longer fever duration and macrolide use (16), while Kim reported that fever duration after initiation of macrolides and corticosteroid treatment was higher in the MRMP population (17), but these two studies only focused on Korean children.

Therefore, this study aims to compare the therapeutic effects of macrolides in MRMP and macrolide-susceptible MP (MSMP) children, providing evidence to guide treatment decisions for pediatric MP infections.

## Materials and methods

### Study design and participants

This retrospective study included pediatric MPP patients who were admitted to the Department of Pediatrics at the hospital between January 2023 and December 2023. The inclusion criteria were: 1) patients who completed targeted next-generation sequencing (tNGS) for upper respiratory tract pathogens and showed positive MP DNA; 2) patients diagnosed with MPP according to the diagnostic criteria in the Guidelines for the Diagnosis and Treatment of Mycoplasma Pneumoniae Pneumonia in Children (MPP Guidelines) (18), the detailed diagnostic criteria were: I) The single serum specific MP antibody titer ≥1:160 (gelatin particle agglutination assay); or during the disease, the double serum specific MP antibody titer increased by 4 times or more; II) positive MP-DNA or MP-RNA. The exclusion criteria were: 1) patients with confirmed viral, bacterial or other pathogen infections, as well as those with non-infectious causes of fever such as Kawasaki disease; 2) Fever resulting from non-respiratory infections; 3) Underlying diseases such as congenital immunodeficiency, severe respiratory and circulatory system disorders, chronic lung diseases, and connective tissue disorders; 4) Long-period users of systemic hormones or immunosuppressants; 5) Individuals allergic to azithromycin, erythromycin, glucocorticoids, etc or with incomplete medical records/self-discharged. This study was granted by the Ethics Committee [Ethical Approval (2025) No. 66], and informed consents were waived due to the retrospective nature of this study.

### Throat swab sampling

On the day of admission, the patient rinsed their mouth with normal saline, and a throat swab was utilized to gently wipe the posterior pharyngeal wall and bilateral tonsils at least 3 times. The swab was rotated to increase the contact area but avoid contact with the tongue surface and oral mucosa. After sampling, the swab was immediately placed into a virus sampling tube containing 3 mL of preservation buffer and transported at 2–8 ℃ within 48 h (on dry ice at −78 ℃) to the laboratory. The KS608-100HXD96 respiratory 100 premix kit (KingMed Diagnostics Group Co., Guangzhou, China) and KM MiniSeqDx-CN library system (KingMed Diagnostics Group Co., Guangzhou, China) were used to amplify and enrich the genes of target pathogenic microorganisms for further gene sequencing. High-throughput sequencing was carried out using the NGS technique to detect the presence of macrolide resistance genes in MP. Cases with positive drug resistance genes (A2063G, A2063T, or A2064G mutations in the V region of the target 23S rRNA gene) were classified as the MRMP group; while those with negative drug resistance genes were included in the MSMP group.

### Treatment procedure

After throat swab acquisition, all patients enrolled in this research received standard treatment with macrolides; specifically, all patients received intravenous injections of 30–45 mg/kg/d erythromycin for a consecutive 7–14 days. The dosage and duration of medication should be adjusted appropriately based on clinical conditions and the physician's judgment. Glucocorticoids and condition-based bronchofibroscopic intervention treatment were administered based on the number of affected lung lobes, extent/density of pulmonary consolidation, CRP and lactate dehydrogenase levels, and performance according to clinicians' experience (18).

Bronchoalveolar lavage fluid (BALF) sampling was performed only in patients with clinical indications for bronchofibroscopy, including extensive pulmonary consolidation, persistent fever >72 h, or poor initial response to macrolide therapy, as determined by the attending physician according to the MPP Guidelines (18). Treating clinicians were not aware of the macrolide resistance status (MRMP vs. MSMP) at the time of BALF sampling, as tNGS results were typically available after the procedure. And this resulted in BALF samples from 29 patients (13.4% of the total cohort).

### Data collection and definition

Baseline characteristics, including admission date, sex, age, clinical symptoms, hematological examination results (including white blood cell count (WBC) and CRP), examination results, treatment protocols, length of hospital stay, duration of fever, utilization of glucocorticoids, and DNA sequencing data, were obtained from the hospital's electronic medical records system.

Macrolide-unresponsive MPP (MUMPP) according to the Guidelines for the Diagnosis and Treatment of Mycoplasma pneumoniae Pneumonia in Children (2025 edition) (19) is defined as an MPP with persistent fever that persists after 72 h of treatment with macrolides, and no improvement or further exacerbation in clinical symptoms and pulmonary imaging.

### Statistical analysis

All statistical analyses were performed using SPSS 23.0 (IBM Corp, New York, USA). The normality of the data was assessed using the Shapiro–Wilk test, continuous variables that met the assumption of normality were presented as mean ± standard deviation (SD), and compared using the independent samples t-test. Variables that did not conform to a normal distribution are expressed as median (interquartile range, IQR) and were compared using the Mann–Whitney U test. Categorical variables were presented in the form of cases and percentages (%) and compared using the Chi-square test. In this study, a two-sided *P* < 0.05 was considered statistically significant.

## Results

### Baseline characteristics

After inclusion and exclusion, a total of 217 patients were included for analysis. Among these patients, 188 (99 male, 5.84 ± 2.81 years) were categorized into the MRMP group, with a prevalence of 86.6%, while 29 (14 male, 6.79 ± 4.08 years) were included in the MSMP group. No significant differences were observed between the two groups in terms of sex distribution (*P* = 0.660), age (*P* = 0.233), or age subgroup distribution (all *P* > 0.05). Similarly, laboratory parameters at admission, including white blood cell count [7.23 (6.08, 9.58) vs. 8.30 (5.60, 9.64), *P* = 0.971], neutrophil percentage [0.64 (0.55, 0.71) vs. 0.56 (0.42, 0.71), *P* = 0.104], lymphocyte percentage [0.28 (0.21, 0.37) vs. 0.23 (0.16, 0.37), *P* = 0.102], and C-reactive protein [11.01 (4.94, 21.99) vs. 7.70 (2.99, 23.24), *P* = 0.501], were comparable between the MRMP and MSMP groups. In addition, no statistically significant differences were found in imaging manifestations at admission (*P* = 0.833), including interstitial lesions, mixed lesions, consolidated lesions, and pleural effusion (all *P* > 0.05) (Table 1).

**Table 1:** Comparison of demographics and clinical data before macrolide use between MRMP and MSMP patients.

Variables	MRMP (*n* = 188)	MSMP (*n* = 29)	*U/t/χ^2^*	*P*
Demographics				
Gender, *n* (%)				
Male	99 (52.7%)	14 (48.3%)	0.193	0.660
Female	88 (47.3%)	15 (51.7%)		
Age	5.84 ± 2.81	6.79 ± 4.08	1.215	0.233
Infants (<1 year), *n* (%)	8 (4.3%)	1 (3.4%)	0.000	1.000
Children (1 to 3 years)	34 (18.1%)	8 (27.6%)	1.453	0.310
Preschool children (4 to 6 years)	69 (36.7%)	5 (17.2%)	4.234	0.057
Schoolchildren (7 to 14 years)	77 (41.0%)	15 (51.7%)	1.193	0.315
Laboratory data at admission				
WBC count (×10^9^/L)	7.23 (6.08, 9.58)	8.30 (5.60, 9.64)	2714.500	0.971
Neutrophils (%)	0.64 (0.55, 0.71)	0.56 (0.42, 0.71)	2214.500	0.104
Lymphocytes (%)	0.28 (0.21, 0.37)	0.23 (0.16, 0.37)	2212.000	0.102
CRP (mg/L)	11.01 (4.94, 21.99)	7.70 (2.99, 23.24)	2514.000	0.501
Imaging manifestations at admission, *n* (%)			0.938	0.833
Interstitial lesion	110 (58.5%)	17 (58.6%)	0.000	0.991
Mixed lesion	58 (30.8%)	10 (34.5%)	0.154	0.695
Consolidated lesion	15 (8.0%)	1 (3.45%)	0.237	0.626
Pleural effusion	5 (2.7%)	1 (3.45%)	0.000	1.000

Data were described as mean ± standard deviation or *n* (%) or median (Q1, Q3). MRMP, macrolide-resistant Mycoplasma pneumoniae; MSMP, macrolide-susceptible Mycoplasma pneumoniae; WBC, white blood cell; CRP, C-reactive protein.

### Comparison of MP DNA load between the MRMP and the MSMP groups

All patients in both MRMP and MSMP groups received throat swabs at admission, and the results showed that the MP DNA load was markedly higher in MRMP patients compared to MSMP patients (3.97 ± 0.64 vs. 3.65 ± 0.77; *P* = 0.039). Although only 29 patients (13.4% of the total cohort; 20 MRMP and 9 MSMP) underwent bronchofibroscopic examination due to clinical indications (e.g., severe consolidation or refractory symptoms), the subgroup analysis still showed significantly higher MP DNA load in BALF from MRMP patients (4.11 ± 0.58 vs. 3.18 ± 0.77; *P* = 0.001) (Table 2).

**Table 2:** MP DNA load of MRMP and MSMP patients.

Variables	MRMP		MSMP		*t*	*P*
	*n*	log_10_ MP DNA	*n*	log_10_ MP DNA		
Throat swab at admission	188	3.97 ± 0.64	29	3.65 ± 0.77	−2.148	0.039*
Bronchoalveolar lavage fluid after admission	20	4.11 ± 0.58	9	3.18 ± 0.77	−3.623	0.001*

MP, Mycoplasma pneumoniae; MRMP, macrolide-resistant Mycoplasma pneumoniae; MSMP, macrolide-susceptible Mycoplasma pneumoniae. BALF sampling limited to clinically indicated cases (*n* = 29). *values in bold, *P* < 0.05.

### Comparison of characteristics after macrolide-based anti-infection treatment

No significant differences were observed between the MRMP and MSMP groups in terms of length of hospital stay [7.00 (5.00, 9.00) vs. 7.00 (6.00, 8.00) days, *P* = 0.301] or total duration of fever [9.00 (7.00, 10.00) vs. 8.00 (6.00, 10.00) days, *P* = 0.090]. Laboratory parameters before discharge, including white blood cell count [8.21 (6.35, 10.21) vs. 7.64 (6.17, 12.11), *P* = 0.718], neutrophil percentage [0.57 (0.48, 0.65) vs. 0.54 (0.40, 0.64), *P* = 0.126], lymphocyte percentage [0.33 (0.26, 0.42) vs. 0.39 (0.26, 0.50), *P* = 0.245], and C-reactive protein [2.75 (0.85, 4.92) vs. 3.76 (0.99, 8.13), *P* = 0.170], were also comparable between the two groups. During treatment, no statistically significant differences were found in the incidence of MUMPP [17.0% vs. 17.2%, *P* = 1.000], glucocorticoid utilization [17.2% vs. 23.4%, *P* = 0.460], duration of rales after macrolide use [6.00 (4.00, 7.00) vs. 5.00 (4.00, 8.00) days, *P* = 0.875], or duration of fever after macrolide use [2.00 (1.00, 3.00) vs. 2.00 (1.00, 2.00) days, *P* = 0.210]. Imaging outcomes at 7 days after treatment were not significantly different between the two groups (*P* = 0.051).

However, the duration of macrolide use was significantly longer in the MRMP group compared with the MSMP group [7.00 (7.00, 9.00) vs. 6.00 (5.00, 8.00) days, *P* = 0.001]. In addition, the incidence of severe Mycoplasma pneumoniae pneumonia (SMPP) was 5.8% in the MRMP group and 17.2% in the MSMP group (*P* = 0.071) (Table 3).

**Table 3:** Laboratory data and outcomes between MRMP and MSMP patients after anti-infection with macrolides.

Variables	MRMP (*n* = 188)	MSMP (*n* = 29)	*U/χ^2^*	*P*
Length of stay (day)	7.00 (5.00, 9.00)	7.00 (6.00, 8.00)	2405.500	0.301
Duration of fever (day)	9.00 (7.00,10.00)	8.00 (6.00,10.00)	2196.000	0.090
Laboratory data at recheck before discharge				
WBC count (×10^9^/L)	8.21 (6.35, 10.21)	7.64 (6.17,12.11)	2612.500	0.718
Neutrophils (%)	0.57 (0.48, 0.65)	0.54 (0.40, 0.64)	2245.000	0.126
Lymphocytes (%)	0.33 (0.26, 0.42)	0.39 (0.26, 0.50)	2360.500	0.245
CRP (mg/L)	2.75 (0.85, 4.92)	3.76 (0.99, 8.13)	2294.500	0.170
Manifestations during the macrolide treatment				
MUMPP [*n* (%)]	32 (17.0%)	5 (17.2%)	0.000	1.000
Utilization of glucocorticoids [*n* (%)]	44 (17.2%)	5 (23.4%)	0.546	0.460
Duration of rales after macrolide use (d)	6.00 (4.00,7.00)	5.00 (4.00,8.00)	2677.000	0.875
Duration of fever after macrolide use (d)	2.00 (1.00, 3.00)	2.00 (1.00, 2.00)	2351.000	0.210
Days of macrolide use (d)	7.00 (7.00, 9.00)	6.00 (5.00, 8.00)	1731.000	0.001*
Imaging manifestations 7 days after treatment			5.858	0.051
Complete absorption [*n* (%)]	121 (64.4%)	23 (79.3%)	2.515	0.141
Partial absorption [*n* (%)]	66 (35.1%)	5 (17.2%)	3.643	0.056
Exacerbation [*n* (%)]	1 (0.5%)	1 (3.4%)	/	0.250
SMPP	11 (5.8%)	5 (17.2%)	3.251	0.071

MRMP, macrolide-resistant Mycoplasma pneumoniae; MSMP, macrolide-susceptible Mycoplasma pneumoniae; WBC, white blood cell; CRP, C-reactive protein; SMPP, severe Mycoplasma pneumoniae pneumonia. *values in bold, *P* < 0.05.

## Discussion

This study showed that no significant difference was observed between MRMP and MSMP patients; however, the MP DNA load was higher in both throat swab and bronchoalveolar lavage fluid samples obtained from MRMP patients. As for the characteristics of anti-infection treatment, only the days of macrolide use were higher in MRMP patients.

The MRMP proportion surged from 80.00% to 93.02% in China between 2023 and 2024 (20), which was in alignment with this study's results, 86.6% MPP patients were found to carry drug-resistance genes. The most prevalent mutations involve the substitution of adenine (A) at positions A2063 or A2064 with cytosine (C), guanine (G), or thymine (T) (21). While in China, the A2063G mutation is the predominant genotype, accounting for over 95% of macrolide-resistant M. pneumoniae (MRMP) cases (22). This study also confirmed previous findings; all resistance-associated mutations identified were A2063G. This suggests that while macrolide resistance rates have risen during MP outbreaks, the resistance genotype has remained relatively stable. Although earlier studies have reported higher MP infection rates in females compared to males, potentially attributable to gender-specific hormonal influences (23), this study found no significant age- or gender-based differences in resistance patterns.

An existing study has unveiled that macrolide drugs remain effective in the treatment of MRMP patients (24). The clinical characteristics, laboratory tests, and imaging results of the two groups in our experiments also validated the therapeutic performance of macrolide drugs for MRMP patients. In addition to its direct antibacterial effect on macrolides, the effectiveness is related to the self-limited property of MP infection and the extensive immunomodulatory effects of macrolide antibiotics both *in vivo* and *in vitro*, which alleviate the body's immune inflammatory response (25, 26). Despite consistently higher MP DNA load in the MRMP patients than the MSMP patients at different disease phases, this research suggested that the immunomodulatory roles of macrolide drugs may contribute to improving the clinical symptoms of these MRMP patients. A report shows that high MP DNA load may cause more serious airway damage (27), yet there are also studies showing that MP-resistant strains do not worsen the condition (28), and MRMP infection shares no significant association with the severity and prognosis of lung damage (29). The inconsistency of research results may be related to the fact that most studies are retrospective, and the timing of drug intervention varies.

Interestingly, although the incidence of SMPP was numerically higher in the MSMP group than in the MRMP group, this difference did not reach statistical significance. These findings suggested that macrolide treatment remains effective in both groups, but MRMP patients may require a longer treatment course, and the results should be interpreted cautiously. First, the MSMP group included only 29 patients, among whom 5 developed SMPP; therefore, the relatively small sample size may have introduced sampling variability and limited statistical power, increasing the likelihood of random fluctuation (30). Second, although baseline demographic characteristics and inflammatory markers at admission were comparable between groups, subtle differences in disease progression prior to hospitalization or in host immune response intensity may not have been fully captured by conventional laboratory parameters. Third, accumulating evidence suggests that excessive host immune activation, rather than macrolide resistance genotype alone, plays a dominant role in the development of SMPP (31). Thus, the occurrence of severe disease in MSMP patients further supports the concept that resistance status is not the sole determinant of clinical severity. Larger, multicenter prospective studies are warranted to clarify the relationship between resistance genotype and disease severity.

In this study, the therapeutic schedule for MPP is based on the recommendations of the MPP Guidelines, considering the adverse reactions of intravenous azithromycin to infants and children, the macrolide drugs used in the treatment were intravenous erythromycin. It was observed that MP-DNA load in the respiratory tract was higher and decreased more slowly in the MRMP patients. The duration of macrolide use was also longer, indicating attenuated biological effects and clinical performance of macrolide drugs for these patients. Choi's could partially support these findings, they found the fever time was longer in MRMP children after macrolide initialization (16). An insignificant difference was also noted in the final length of stay and imaging review results, indicating that even in the presence of macrolide-resistant strains, macrolide drugs can continue to be used for treatment, but the duration of use needs to be prolonged, which may be related not only to drug resistance but also to pathogen load.

In addition, glucocorticoids are a battery of steroid hormones released from the adrenal cortex that regulate the nervous and immune systems of the body by binding to glucocorticoid receptors (32), while immune suppression is their main function. Systemic glucocorticoids can reduce the host's immune response and are also available for the treatment of MRMP. The MPP Guidelines recommend the routine use of methylprednisolone 2 mg/kg/d for severe and critically ill patients. Kim reported that corticosteroid use were higher in MRMP population among Korean MPP children, while our research exhibited no statistical difference concerning the use of glucocorticoids as well as no significant difference regarding inflammatory indicators such as WBC count and CRP between the two groups, suggesting the potential regional difference on MRMP severity, another study performed in Northern children also showed no significant difference on WBC and CRP, but the LDH normal rate was lower in MRMP children (33). Moreover, in the study performed by Kim (17), no sequencing was performed; the location of the point mutation could also affect the severity of the MRMP, which required further study.

This study still possesses several limitations, first, this is a single-center study that only includes Chinese children, which may reduce the generalizability of the results; second, although BALF sampling was performed only when clinically indicated and strictly based on local institutional guidelines, bronchoscopy in children with acute Mycoplasma pneumoniae pneumonia remains an invasive procedure with potential risks, which also limited the BALF sample size. The relatively small sample size may introduce potential selection bias toward more severe patients, and the finding of higher MP DNA load in BALF of MRMP patients should be interpreted with caution. In the future, broader geographical coverage, more comprehensive respiratory tract sampling, and multi-center prospective studies should be performed to validate our findings and improve the reliability and clinical application value of research results.

## Conclusions

In conclusion, this study reveals a high incidence of MRMP characterized by the A2063G point mutation in 23S rRNA. The results also showed that MRMP patients carry higher MP DNA load and longer macrolide usage duration. However, MRMP does not increase the incidence of SMPP and other adverse events, indicating macrolides are safe and effective in both MRMP and MSMP.

## Data Availability

The original contributions presented in the study are included in the article/Supplementary Material, further inquiries can be directed to the corresponding author.
